# Accelerated Brain Aging, Atherogenicity, and Neurocognition in Adult Survivors of Childhood Cancer

**DOI:** 10.1001/jamanetworkopen.2025.51865

**Published:** 2025-12-30

**Authors:** Nicholas S. Phillips, Silu Zhang, Jessica Baedke, Qing Ji, Noah D. Sabin, Kirsten K. Ness, Melissa M. Hudson, Yutaka Yasui, Sebabrata Mahapatra, Meenasri Kumbaji, Ruth G. Tatevossian, Matthew A. Scoggins, AnnaLynn M. Williams, Kevin R. Krull

**Affiliations:** 1 Departments of Psychology and Biobehavioral Science, St Jude Children’s Research Hospital, Memphis, Tennessee; 2 Departments of Epidemiology and Cancer Control, St Jude Children’s Research Hospital, Memphis, Tennessee; 3 Department of Oncology, St Jude Children’s Research Hospital, Memphis, Tennessee; 4 Department of Radiology, St Jude Children’s Research Hospital, Memphis, Tennessee; 5 Department of Clinical Biomarkers Laboratory, St Jude Children’s Research Hospital, Memphis, Tennessee; 6 Department of Surgery, Wilmot Cancer Institute, University of Rochester Medical Center, Rochester, New York; 7 Clinical Biomarkers Laboratory, St Jude Children's Research Hospital, Memphis, Tennessee

## Abstract

**Importance:**

Long-term survivors of childhood cancer may be at elevated risk for accelerated brain aging.

**Objectives:**

To determine the brain age gap estimation (BrainAGE), defined as the difference between estimated brain age and chronological age; the association between BrainAGE scores and poor neurocognitive outcomes; and the correlations of plasma biomarkers of oxidative stress, neuroinflammation, and cardiovascular health with older BrainAGE scores and previous exposure to central nervous system–directed therapy in adult survivors of childhood cancer.

**Design, Setting, and Participants:**

This cross-sectional study analyzed participants in the St. Jude Lifetime Cohort Study. Included participants were survivors of childhood cancer (brain tumor, acute lymphoblastic leukemia, and Hodgkin lymphoma) and community controls (no history of childhood cancer) aged 18 years or older with a whole brain scan following a neurocognitive assessment. BrainAGE was calculated from January 2016 to February 2021 and analyzed from February 2022 to March 2025. Treatment information, plasma biomarkers, and neurocognitive outcomes were collected.

**Main Outcomes and Measures:**

The primary outcome was estimated brain age (using previously trained machine learning pipelines) and associations of brain age with neurocognitive outcomes. Associations among BrainAGE scores, plasma biomarkers, and neurocognitive outcomes were examined using multivariable linear regression models and Spearman correlations.

**Results:**

A total of 253 survivors of childhood cancer (127 males [50.2%]; mean [SD] age, 31.7 [8.6] years; mean [SD] time since diagnosis, 21.2 [9.8] years) and 43 community controls (24 females [55.8%]; mean [SD] age at evaluation, 31.4 [9.6] years) were included in analyses. Survivors had significantly higher mean BrainAGE scores than controls (6.6 [95% CI, –12.5 to 41.1] years vs 0.7 [95% CI, –1.0 to 2.4] years older than chronological age; *P* < .001). A 10-year increase in BrainAGE score was associated with lower cognitive flexibility (β = −0.63; 95% CI, −0.82 to −0.45), processing speed (β = −0.49; 95% CI, −0.67 to −0.31), working memory (β = −0.28; 95% CI, −0.53 to −0.03) and visual memory (β = −0.40; 95% CI, −0.58 to −0.22), vocabulary (β = −0.33; 95% CI, −0.48 to −0.18), and reading (β = −0.31; 95% CI, −0.45 to −0.18) *z* scores after adjusting for age at diagnosis and sex. Neurofilament light was correlated with BrainAGE score in female survivors (Spearman ρ = 0.24 [*P* = .04]) and controls (Spearman ρ = −0.47 [*P* = .03]). Female survivors who were younger than 10 years at cancer diagnosis and treated with 40 Gy or higher cranial radiation had mean BrainAGE scores higher than 30 years (37.34 [95% CI, 17.4-41.0] years) older than chronological age (*P* = .004) and had lower sex hormone precursor correlated with higher BrainAGE score (Spearman ρ = −0.44 [*P* = .01]) and higher cranial radiation dose (Spearman ρ = −0.48 [*P* = .01]). Among male survivors, correlations of BrainAGE score and cranial radiation dose with malondialdehyde (Spearman ρ = −0.25 [*P* = .04] and −0.26 [*P* = .04]) and oxidized low-density lipoprotein (Spearman ρ = 0.24 [*P* = .049] and 0.40 [*P* = .001]) were identified.

**Conclusion and Relevance:**

This cross-sectional study found that accelerated brain aging was associated with poorer neurocognitive outcomes and correlated with cranial radiation, biomarkers of atherogenesis, neuronal damage, and sex hormone precursors. These findings can guide future research efforts to reduce the risk of vascular disease in this at-risk population.

## Key Points

**Question:**

Do survivors of childhood cancer experience accelerated brain aging associated with neurocognitive impairments, biomarkers of oxidative stress, and neuroinflammation?

**Findings:**

In this cross-sectional study of 253 survivors of childhood cancer and 43 community controls, survivors had statistically significant accelerated brain aging (6.6 years older) compared with controls, which was associated with lower neurocognitive function and elevated biomarkers of oxidative stress, vascular health, and neuroinflammation. Female survivors who were treated with 40 Gy or higher cranial radiation before 10 years of age had the most evidence of brain aging.

**Meaning:**

These findings may guide future research efforts to reduce the risk of vascular disease in this at-risk population.

## Teaser

This cross-sectional study examines the association between brain aging and neurocognitive outcomes in survivors of childhood cancer and individuals without childhood cancer history.

## Introduction

Improvements in cancer therapy have led to a growing population of nearly 500 000 aging adult survivors of childhood cancer in the US.^1^ Research demonstrates that cancer survivors are at risk for accelerated neurocognitive declines.^2,3^ A recent study using a large well-characterized cohort documented that adult survivors of childhood cancer without baseline neurocognitive problems reported greater symptoms of neurocognitive decline than their siblings over an 11-year period.^4^ Memory impairment was the most reported problem, even among those who received no central nervous system (CNS)-directed therapy, and may signal that this population is at risk for treatment-related early-onset mild cognitive impairment or dementia. However, few biomarkers exist that can identify who may carry the highest risk and what underlying etiological factors may be implicated.

Normal brain aging is associated with nonlinear changes in gray and white matter volumes over time.^5^ Deviations from normal brain aging in populations without cancer are associated with neurologic and neurocognitive impairments.^6^ Brain age gap estimation (BrainAGE) is a machine learning method used to compare clinically obtained structural magnetic resonance imaging (MRI) with an artificial intelligence–generated reference brain aging model.^7^ A BrainAGE score is the difference between an individual’s brain age, estimated from the brain’s morphological structure, and their chronological age. Positive BrainAGE scores indicate an older estimated brain age than chronological age. In populations without cancer, phenotypic older-appearing brains (positive BrainAGE scores from 2 to 8 years) are associated with the development of mild cognitive impairments, Alzheimer disease, and even premature death.^8,9^ However, evidence of similar associations with advanced structural brain aging in adult childhood cancer survivors has not been reported. BrainAGE differences may be a risk factor associated with poor neurocognitive outcomes and may aid in the identification of clinically vulnerable survivors in need of interventions.

Among this at-risk population are survivors of childhood brain tumor, acute lymphoblastic leukemia (ALL), and Hodgkin lymphoma who demonstrate elevated plasma biomarkers of inflammation and oxidative stress decades after completion of cancer therapy.^10,11^ Methotrexate, commonly used for the treatment of ALL, has been associated with oxidative stress and leukoencephalopathy, while cranial radiation and neurosurgery have been associated with biomarkers of inflammation in the cerebrospinal fluid of a small sample of survivors of medulloblastoma. Moreover, chest radiation has been associated with increased risk for cardiovascular disease and neurocognitive impairment in long-term survivors of childhood cancer.^12^ Sex differences have also been reported in the neurocognitive outcomes of childhood cancer survivors, and sex hormones are known to be neuroprotective against brain aging in populations without cancer.^13^ However, it is unknown whether sex hormones are protective among survivors or whether elevated levels of markers of systemic inflammation are associated with accelerated structural brain aging. This research provides critical information about the pathophysiological characteristics of neurocognitive declines. The aim of this study was to determine BrainAGE; the association between BrainAGE scores and poor neurocognitive outcomes; and the correlations of plasma biomarkers of oxidative stress, neuroinflammation, and cardiovascular health with older BrainAGE scores and previous exposure to CNS-directed therapy in adult survivors of childhood cancer.

## Methods

Survivors and community controls analyzed in this cross-sectional study were recruited from the St. Jude Lifetime Cohort Study, a retrospectively identified cohort with longitudinal follow-up of childhood cancer survivors diagnosed at St. Jude Children’s Research Hospital between 1962 and 2012 to assess long-term cancer treatment–related morbidities and mortalities.^14^ The St Jude Children’s Research Hospital Institutional Review Board approved this study. All participants provided written informed consent. We followed the Strengthening the Reporting of Observational Studies in Epidemiology (STROBE) reporting guideline.

Survivors of ALL, Hodgkin lymphoma, and brain tumor (CNS) and controls were included in this study if they were 18 years or older and had a noncontrast 3-dimensional (3-D) T1-weighted MRI whole brain scan within 180 days of completion of a 2-hour neurocognitive assessment. Survivors and controls who were diagnosed with a genetic disorder that predisposes neurocognitive decline not related to cancer (eg, Trisomy 21 and Turner syndrome) were excluded from this study. In the St. Jude Lifetime Cohort Study, community controls are recruited based on age, sex, and race frequency matching. They cannot be first degree relatives of survivors, cannot have a history of childhood cancer, and cannot be pregnant. Otherwise, they are not excluded due to their health history. Controls are recruited from the same geographic and socioeconomic populations as the survivors.

### Neurocognitive Assessment

Neurocognitive testing used measures of intelligence (Wechsler Abbreviated Scale of Intelligence [WAIS-4]^15^); word reading (Woodcock-Johnson Tests of Cognitive Abilities); sustained attention (Conner Continuous Performance Test-II); memory (California Verbal Learning Test, second edition^16^); visual memory (Visual Selective Reminding Test Board); processing speed (Digit-Symbol Coding Test^15^ and Grooved Pegboard Test^17,18^); and executive function, including cognitive flexibility (Trail Making Test,^19^ Delis-Kaplan Executive Function System Verbal Fluency Test,^20^ and WAIS-4 Digit Span) (eTable 1 in Supplement 1). Raw scores were converted into age-adjusted *z* scores based on population normative data. Participants with missing neurocognitive variables were excluded from the analysis.

### Neuroimaging

BrainAGE was calculated from January 2016 to February 2021 using open-source Gaussian process regression software (BrainAgeR) on visually inspected, segmented, and normalized, noncontrast, 3-D T1-weighted MRI scans.^21,22^ To compare the BrainAGE scores of survivors and controls, we performed a Wilcoxon rank sum test from September 2022 to January 2024.

### Biomarker Assay

Blood was collected via venous draw within 90 days of MRI examination, and whole blood was processed for serum stored in 1-mL aliquots at −80° C. Samples were thawed and prealiquoted to accommodate all subsequent downstream testing. Malondialdehyde was measured directly after the initial thawing of the specimens. All other biomarker testing was performed after samples went through 2 freeze-thaw cycles. Interferon γ, interleukin 10 (IL-10), IL-12p70, IL-13, IL-1B, IL-2, IL-4, IL-6, IL-8, and tumor necrosis factor (TNF) were measured using an immunoassay kit (V-PLEX Plus Proinflammatory Panel 1; Meso Scale Discovery). Plate-based electrochemiluminescence immunoassay platform (Meso Scale Discovery) was also used to measure high-sensitivity C-reactive protein (CRP), N-terminal pro–brain natriuretic peptide, neurofilament light, and soluble TNF receptor I (sTNFR-I) and II (sTNFR-II). Samples were tested for oxidized low-density lipoprotein (LDL), 8-hydroxyguanosine, and dehydroepiandrosterone sulfate (DHEA-S) using enzyme-linked immunosorbent assays (ELISA; Mercodia Uppsala, Cell Biolabs, and Abcam). Thiobarbituric acid reactive substances assay (Cell Biolabs) was used to estimate malondialdehyde. Glutathione peroxidase and superoxide dismutase activities were measured by enzymatic assays (R&D Systems). An enzyme-based homocysteine assay was used (Diazyme Laboratories).

All assays were performed following their manufacturer’s instructions, samples were run at least in duplicate, and concentrations of analytes were interpolated from standard curves. Intra-assay and interassay coefficients of variation were less than 20.0%. To estimate values below the reliable detection limit for each biomarker assay, we performed a stochastic imputation using a random number generator bounded by the lower limit of detection and 0. This process was repeated 20 times to create multiple imputation sets. The final imputation value was estimated as the mean value across these 20 sets.

We also tested samples for 8-iso-prostaglandin F2α, 3-Nitrotyrosine, and 4-hydroxynonenal using ELISA kits (Cell Biolabs). The mean intra-assay and interassay coefficients of variation for these assays were more than 20.0%. These assays were deemed unsuitable for the purpose and were discontinued.

### Statistical Analysis

Descriptive statistics were generated using χ^2^ for categorical variables and an unpaired, 2-tailed *t* test for continuous variables. Benjamini-Hochberg–adjusted *t* tests were used to compare age-adjusted neurocognitive *z* scores between controls and survivors. A similar analysis was undertaken to compare BrainAGE scores between controls and survivors. Linear regression estimated associations of brain age acceleration (brain age minus chronological age), treatments, and neurocognitive outcomes with models adjusted for age at diagnosis and sex in survivors. Multivariable linear regressions were used to further evaluate whether high-dose methotrexate and intrathecal methotrexate, chest radiation, and/or cranial radiation interactions were associated with BrainAGE, adjusting for age at diagnosis and sex. To assess whether neurosurgical procedures, such as tumor resection or shunt placement, affected BrainAGE scores, we assessed the distribution of BrainAGE scores among brain tumors with supratentorial vs infratentorial location and presence of shunt. Multivariable linear regression models were used to assess the association of cranial radiation dose in the brain (maximum dose: 2-19 Gy, 20-39 Gy, and ≥40 Gy compared with 0 or scatter only [0.2 Gy]) with BrainAGE scores, adjusting for age at diagnosis, time since diagnosis, sex, and chemotherapy exposures. Because treatment with cranial radiation is delayed in children diagnosed before the age of 3 years, associations among BrainAGE, biomarkers, and neurocognitive outcomes were explored using multivariable models, controlling for age at treatment instead of age at diagnosis in that subpopulation. Exploratory comparisons between BrainAGE scores, plasma biomarkers, and radiation dose were conducted using Spearman correlations (Spearman ρ) without multiple comparison corrections, adjusting for sex and, for survivors, age at diagnosis.

Two-sided *P* < .05 indicated statistical significance. Data analysis was performed from July 2022 to March 2025 using R, version 4.4.2 (R Project for Statistical Computing).

## Results

**Figure 1.**
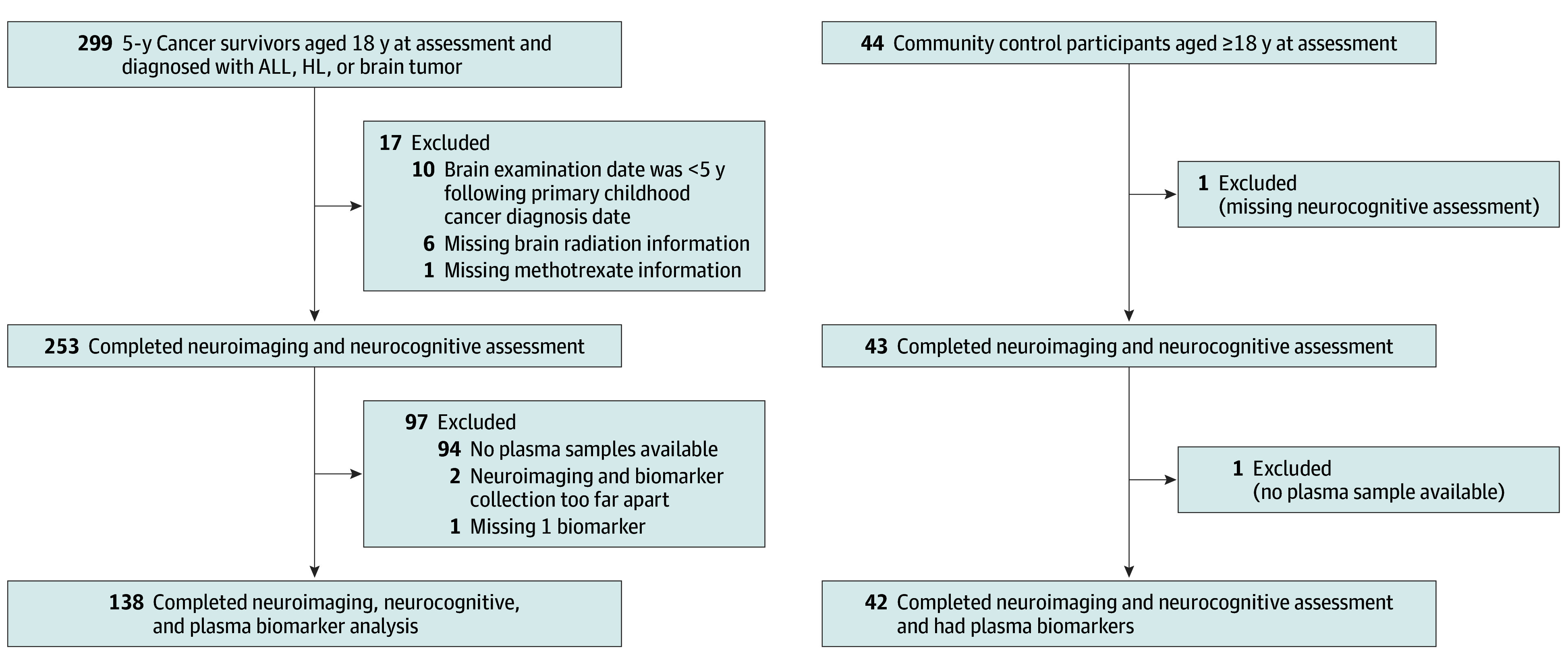
CONSORT Diagram of Survivors of Childhood Cancer ALL indicates acute lymphoblastic leukemia; HL, Hodgkin lymphoma.

A total of 253 childhood cancer survivors (ALL [118], brain tumor [49], and Hodgkin lymphoma [86]) and 43 community controls were included in this study (Figure 1). Among survivors, 126 (49.8%) were females and 127 (50.2%) were males with a mean (SD) age of 31.7 (8.6) years at MRI and neurocognitive assessment and a mean (SD) time since diagnosis of 21.2 (9.8) years. The 43 controls (24 females [55.8%]) had a mean (SD) age of 31.4 (9.6) years at time of evaluation (Table 1).

**Table 1.** Participant Demographic, Diagnosis, and Treatment Characteristics

Characteristic	Community controls, No. (%) (n = 43)				Cancer survivors, No. (%) (n = 253)				*P* value
	All	Male	Female	*P* value	All	Male	Female	*P* value	
All	43 (100)	19 (44.2)	24 (55.8)	NA	253 (100)	127 (50.2)	126 (49.8)	NA	.57
Age at MRI and neurocognitive assessment, mean (SD), y	31.4 (9.6)	31.7 (10.0)	31.2 (9.4)	.87	31.7 (8.6)	31.5 (8.9)	32.0 (8.3)	.66	.83
Age at diagnosis, mean (SD), y	NA	NA	NA	NA	10.1 (5.4)	9.8 (5.3)	10.4 (5.4)	.38	NA
Time since diagnosis, mean (SD), y	NA	NA	NA	NA	21.2 (9.8)	21.2 (10.3)	21.2 (9.4)	>.99	NA
Brain tumor	NA	NA	NA	NA	49 (19.4)	33 (26.0)	16 (12.7)	NA	NA
Infratentorial	NA	NA	NA	NA	31 (12.3)	23 (18.1)	10 (7.9)	NA	NA
Supratentorial	NA	NA	NA	NA	14 (5.5)	9 (7.1)	7 (5.6)	NA	NA
Other^a^	NA	NA	NA	NA	4 (1.6)	4 (3.1)	2 (1.6)	NA	NA
ALL	NA	NA	NA	NA	118 (46.6)	56 (44.1)	62 (49.2)	NA	NA
Hodgkin lymphoma	NA	NA	NA	NA	86 (34.0)	38 (29.9)	48 (38.1)	NA	NA
Cranial radiation	NA	NA	NA	NA	129 (51.0)	70 (55.1)	59 (46.8)	NA	NA
Methotrexate	NA	NA	NA	NA	112 (44.3)	52 (40.9)	60 (47.6)	NA	NA
Shunt	NA	NA	NA	NA	8 (3.2)	6 (4.7)	2 (1.6)	NA	NA
Plasma biomarkers collected	42 (97.7)	19 (44.2)	23 (95.8)	NA	138 (54.5)	68 (53.5)	70 (55.6)	NA	.78
Brain tumor	NA	NA	NA	NA	14 (5.5)	10 (7.9)	4 (3.2)	NA	NA
ALL	NA	NA	NA	NA	53 (20.9)	24 (18.9)	29 (23.0)	NA	NA
Hodgkin lymphoma	NA	NA	NA	NA	71 (28.1)	34 (26.8)	37 (29.4)	NA	NA

Abbreviations: ALL, acute lymphoblastic leukemia; MRI, magnetic resonance imaging; NA, not applicable.

^a^ Other includes both infratentorial and supratentorial or extra-axial tumors; these were not differentiated for this analysis.

**Figure 2.**
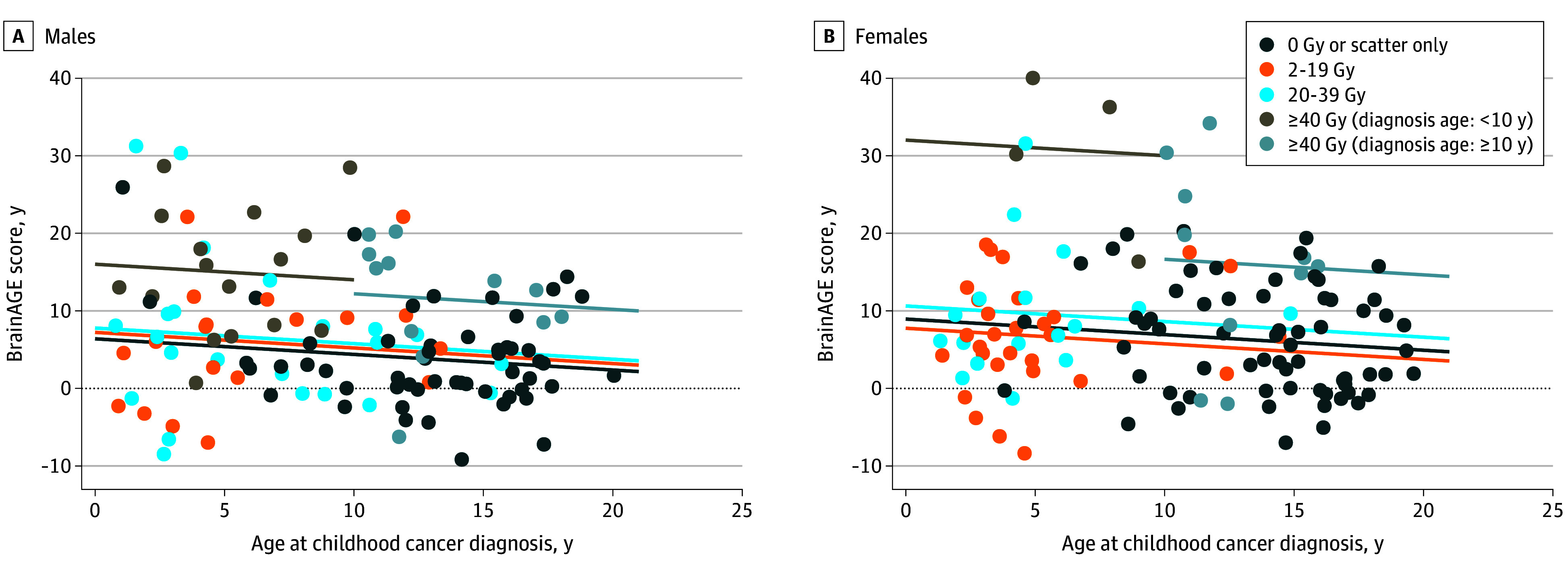
Scatterplot of BrainAGE Score vs Age at Childhood Cancer Diagnosis Stratified by Sex and Cranial Radiation Dose The greatest difference between brain age and chronological age was seen in survivors treated with high-dose cranial radiation compared with those who received no radiation. Female survivors treated with 40 Gy or higher cranial radiation and who were diagnosed before age 10 years had the worse outcomes, with a mean (SD) difference between brain age and actual age of 37.34 (95% CI, 17.4-41.0) years.

Survivors had significantly higher mean BrainAGE scores compared with community controls (6.6 [95% CI, –12.5 to 41.1] years vs 0.7 [95% CI, –1.0 to 2.4] years older than chronological age; *P* < .001). Female survivors who were 10 years or older at cancer diagnosis and treated with 40 Gy or higher cranial radiation dose had mean BrainAGE scores higher than 15 years (16.5 [95% CI, –1.2 to 34.3] years) older than chronological age (*P* = .002). Female survivors who were younger than 10 years at diagnosis had mean BrainAGE scores higher than 30 years (37.34 [95% CI, 17.4-41.0] years) older than chronological age (*P* = .004) (Figure 2).

Compared with controls, survivors had significantly lower mean (SD) *z* scores in word reading (survivors: −0.43 [0.82] vs controls: −0.05 [0.60]; *P* < .001), verbal reasoning (−0.43 [1.14] vs 0.14 [0.93]; *P* < .001), full scale IQ (−0.27 [1.08] vs 0.22 [0.89]; *P* = .002), visual memory (−0.73 [1.29] vs −0.10 [1.03]; *P* = .001), fluency (−0.30 [1.12] vs 0.40 [1.18]; *P* < .001), flexibility (−0.72 [1.72] vs 0.32 [1.03]; *P* < .001), focused attention (−0.15 [1.43] vs 0.51 [0.85]; *P* < .001), motor speed (−0.77 [1.35] vs 0.05 [0.95]; *P* < .001), attention span (−0.37 [1.05] vs 0.13 [0.98]; *P* = .003), working memory (−0.45 [1.09] vs 0.08 [0.75]; *P* = .03), new learning (−0.14 [1.24] vs 0.32 [1.12]; *P* = .02), attention variability (−0.22 [1.15] vs 0.29 [0.99]; *P* = .004), sustained attention (−0.11 [1.17] vs 0.25 [0.68]; *P* = .006), and visuomotor speed (−0.38 [1.07] vs 0.53 [0.90]; *P* < .001) (eFigure 1 and eTable 2 in Supplement 1). A 10-year increase in BrainAGE score was associated with 0.63-SD worse cognitive flexibility (β = −0.63; 95% CI, −0.82 to −0.45; *P* < .001), 0.49-SD poorer motor processing speed (β = −0.49; 95% CI, −0.67 to −0.31; *P* < .001), 0.28-SD lower working memory (β = −0.28; 95% CI, −0.53 to −0.03; *P* = .03) and 0.40-SD lower visual memory (β = −0.40; 95% CI, −0.58 to −0.22; *P* < .001); 0.33-SD worse vocabulary (β = −0.33; 95% CI, −0.48 to −0.18; *P* < .001); and 0.31-SD poorer reading (β = −0.31; 95% CI, −0.45 to −0.18; *P* < .001) *z* scores after adjusting for age at diagnosis and sex (eFigure 2 and eTable 2 in Supplement 1).

BrainAGE was not significantly associated with high-dose intrathecal methotrexate in survivors of ALL who did not receive cranial radiation. However, intrathecal methotrexate was inversely associated with BrainAGE in survivors who received cranial radiation but did not receive high-dose methotrexate (β [SE] = −2.32 [1.02]; *P* = .02). Chest radiation in female survivors of Hodgkin lymphoma was associated with lower (younger) mean BrainAGE scores (β [SE] = −4.88 [2.46]; *P* = .048). Survivors of CNS tumor with infratentorial tumors had higher (older) mean BrainAGE scores than survivors with supratentorial tumors (20.2 [95% CI, 13.5-25.3] vs 11.9 [95% CI, 4.8-17.1] years). Seven of the 8 patients with shunts had infratentorial tumors. However, the presence of a shunt was associated with a lower mean BrainAGE score compared with no shunt (16.5 [95% CI, 8.1-22.8] vs 19.7 [95% CI, 13.5-25.1] years).

Among male survivors, oxidized LDL (Spearman ρ = 0.24 [*P* = .049] and 0.40 [*P* = .001]), malondialdehyde (Spearman ρ = −0.25 [*P* = .04] and −0.26 [*P* = .04]), and sTNFR-II (Spearman ρ = 0.27 [*P* = .03] and 0.27 [*P* = .03]) were directly correlated with both BrainAGE score (Table 2) and cranial radiation dose (Table 2 and Table 3). This correlation differed notably for male controls, among whom only homocysteine (Spearman ρ = 0.61; *P* = .01) was directly correlated with BrainAGE score. Among female survivors and controls, neurofilament light was directly correlated with BrainAGE score (Spearman ρ = 0.24 [*P* = .04] and −0.47 [*P* = .03]). Cranial radiation dose was inversely correlated with 8-hydroxyguanosine (Spearman ρ = −0.37; *P* = .002) and directly correlated with oxidized LDL (Spearman ρ = 0.69; *P* < .001), superoxide dismutase (Spearman ρ = 0.61; *P* < .001), and sTNFR-I and sTNFR-II (Spearman ρ = 0.41 [*P* < .001] and 0.43 [*P* < .001]) in female survivors.

**Table 2.** Spearman Correlations Between Plasma Biomarkers and BrainAGE Score^a^

Biomarker	No. of participants	Mean (SE)	Spearman ρ	*P* value
**Male survivors**				
8-Hydroxyguanosine, ng/mL	68	10.65 (4.57)	−0.15	.21
DHEA-S, μg/dL	68	2.37 (1.33)	−0.10	.41
Glutathione peroxidase, U/mL	68	327.33 (67.97)	−0.07	.57
Homocysteine, μM	68	12.41 (5.44)	−0.22	.08
High-sensitivity CRP, mg/dL	68	3.92 × 10^−7^ (4.50 × 10^−7^)	0.01	.94
Interferon γ, pg/mL	68	6.92 (5.37)	0.25	.04
IL-10, pg/mL	68	0.35 (0.41)	0.15	.22
IL-12p70, pg/mL	68	0.14 (0.20)	0.23	.06
IL-6, pg/mL	68	1.39 (3.23)	−0.09	.47
IL-8, pg/mL	68	16.77 (63.59)	−0.07	.56
Malondialdehyde, μM	68	5.03 (1.19)	−0.25	.04
Neurofilament light, pg/mL	68	58.41 (84.49)	0.03	.81
NT-proBNP, pg/mL	68	396.98 (708.18)	0.02	.89
Oxidized LDL, U/L	68	48.06 (36.18)	0.24	.049
Superoxide dismutase, U/uL	68	0.19 (0.07)	0.20	.11
sTNFR-I, pg/mL	68	965.18 (233.49)	0.07	.55
sTNFR-II, pg/mL	68	7741.10 (2151.01)	0.27	.03
TNF, pg/mL	68	1.66 (0.93)	0.00	.97
**Male controls**				
8-Hydroxyguanosine, ng/mL	19	12.41 (2.71)	0.04	.86
DHEA-S, μg/dL	19	3.03 (1.25)	0.24	.33
Glutathione peroxidase, U/mL	19	318.11 (29.74)	0.03	.91
Homocysteine, μM	19	12.56 (4.71)	0.61	.01
High-sensitivity CRP, mg/dL	19	1.72 × 10^−7^ (1.94 × 10^−7^)	−0.15	.53
Interferon γ, pg/mL	19	5.48 (4.04)	−0.12	.62
IL-10, pg/mL	19	0.28 (0.10)	0.02	.93
IL-12p70, pg/mL	19	0.06 (0.06)	0.23	.34
IL-6, pg/mL	19	3.19 (12.62)	−0.13	.60
IL-8, pg/mL	19	67.34 (269.81)	−0.06	.80
Malondialdehyde, μM	19	5.39 (0.78)	0.13	.59
Neurofilament light, pg/mL	19	31.76 (13.86)	0.30	.21
NT-proBNP, pg/mL	19	154.04 (124.13)	0.15	.54
Oxidized LDL, U/L	19	12.57 (7.63)	0.00	.99
Superoxide dismutase, U/uL	19	0.14 (0.06)	−0.20	.40
sTNFR-I, pg/mL	19	932.17 (191.05)	0.01	.98
sTNFR-II, pg/mL	19	6980.01 (2400.96)	0.32	.19
TNF, pg/mL	19	1.88 (2.32)	0.06	.82
**Female survivors**				
8-Hydroxyguanosine, ng/mL	70	10.54 (9.70)	0.07	.58
DHEA-S, μg/dL	70	1.66 (1.16)	−0.06	.62
Glutathione peroxidase, U/mL	70	314.78 (82.16)	0.19	.11
Homocysteine, μM	70	11.04 (3.03)	0.13	.29
High-sensitivity CRP, mg/dL	70	6.66 × 10^−7^ (7.36 × 10^−7^)	0.22	.07
Interferon γ, pg/mL	70	12.87 (22.45)	0.01	.93
IL-10, pg/mL	70	0.34 (0.27)	−0.05	.68
IL-12p70, pg/mL	70	0.14 (0.16)	0.12	.34
IL-6, pg/mL	70	1.15 (1.31)	0.07	.57
IL-8, pg/mL	70	6.69 (5.15)	−0.03	.83
Malondialdehyde, μM	70	4.82 (0.95)	0.13	.29
Neurofilament light, pg/mL	70	50.41 (64.19)	0.24	.04
NT-proBNP, pg/mL	70	350.74 (385.30)	0.09	.47
Oxidized LDL, U/L	70	39.98 (34.06)	0.08	.53
Superoxide dismutase, U/uL	70	0.21 (0.06)	0.10	.40
sTNFR-I, pg/mL	70	907.21 (250.51)	−0.09	.43
sTNFR-II, pg/mL	70	7219.38 (2311.61)	0.01	.97
TNF, pg/mL	70	1.36 (0.50)	0.05	.67
**Female controls**				
8-Hydroxyguanosine, ng/mL	23	10.30 (3.14)	0.00	.99
DHEA-S, μg/dL	23	1.61 (0.93)	0.00	.98
Glutathione peroxidase, U/mL	23	291.32 (45.68)	−0.21	.34
Homocysteine, μM	23	10.85 (3.39)	−0.03	.89
High-sensitivity CRP, mg/dL	23	5.36 × 10^−7^ (6.75 × 10^−7^)	0.25	.25
Interferon γ, pg/mL	23	12.82 (25.26)	0.00	.99
IL-10, pg/mL	23	0.65 (1.98)	0.11	.62
IL-12p70, pg/mL	23	0.08 (0.13)	0.28	.20
IL-6, pg/mL	23	0.57 (0.50)	0.13	.57
IL-8, pg/mL	23	6.06 (3.18)	0.24	.28
Malondialdehyde, μM	23	4.45 (0.73)	−0.17	.43
Neurofilament light, pg/mL	23	34.49 (25.35)	−0.47	.03
NT-proBNP, pg/mL	23	309.69 (191.33)	0.42	.049
Oxidized LDL, U/L	23	−34.56 (210.25)	0.43	.04
Superoxide dismutase, U/uL	23	0.15 (0.04)	0.10	.65
sTNFR-I, pg/mL	23	958.40 (218.35)	−0.15	.49
sTNFR-II, pg/mL	23	7038.21 (2203.54)	0.22	.31
TNF, pg/mL	23	1.25 (0.35)	−0.25	.24

Abbreviations: BrainAGE, brain age gap estimation; CRP, C-reactive protein; DHEA-S, dehydroepiandrosterone sulfate; IL, interleukin; LDL, low-density lipoprotein; NT-proBNP, N-terminal pro–brain natriuretic peptide; sTNFR, soluble tumor necrosis factor receptor; TNF, tumor necrosis factor.

SI conversion factors: To convert C-reactive protein to milligram per liter, multiply by 10; DHEA-S to micromole per liter, multiply by 0.027; NT-proBNP to nanograms per liter, multiply by 1.

^a^ Higher BrainAGE score indicates greater gap between estimated brain age and chronological age.

**Table 3.** Spearman Correlations Between Plasma Biomarkers and Cranial Radiation Dose in Gy

Biomarker	No.	Mean (SE)	Spearman ρ	*P* value
**Male survivors**				
8-Hydroxyguanosine, ng/mL	65	10.53 (4.51)	−0.28	.02
DHEA-S, μg/dL	65	2.40 (1.35)	−0.20	.10
Glutathione peroxidase, U/mL	65	329.89 (68.44)	0.01	.95
Homocysteine, μM	65	12.36 (5.49)	−0.19	.12
High-sensitivity CRP, mg/dL	65	3.9 × 10^−7^ (4.56 × 10^−7^)	−0.09	.45
Interferon γ, pg/mL	65	6.68 (5.31)	0.09	.47
IL-10, pg/mL	65	0.36 (0.41)	0.23	.06
IL-12p70, pg/mL	65	0.14 (0.21)	0.14	.28
IL-6, pg/mL	65	1.37 (3.30)	−0.12	.32
IL-8, pg/mL	65	16.81 (64.96)	−0.02	.89
Malondialdehyde, μM	65	5.02 (1.21)	−0.26	.04
Neurofilament light, pg/mL	65	58.16 (86.37)	0.13	.31
NT-proBNP, pg/mL	65	349.54 (604.21)	−0.03	.81
Oxidized LDL, U/L	65	48.89 (36.24)	0.40	.001
Superoxide dismutase, U/uL	65	0.19 (0.07)	0.33	.01
sTNFR-I, pg/mL	65	956.54 (229.11)	0.19	.12
sTNFR-II, pg/mL	65	7670.45 (2174.49)	0.27	.03
TNF, pg/mL	65	1.65 (0.94)	0.04	.74
**Female survivors**				
8-Hydroxyguanosine, ng/mL	68	10.37 (9.76)	−0.37	.002
DHEA-S, μg/dL	68	1.67 (1.16)	−0.15	.23
Glutathione peroxidase, U/mL	68	315.79 (82.32)	0.23	.06
Homocysteine, μM	68	11.06 (3.08)	−0.09	.49
High-sensitivity CRP, mg/dL	68	6.75 × 10^−7^ (7.45 × 10^−7^)	0.19	.12
Interferon γ, pg/mL	68	13.03 (22.76)	−0.02	.86
IL-10, pg/mL	68	0.34 (0.27)	0.06	.61
IL-12p70, pg/mL	68	0.14 (0.17)	0.07	.60
IL-6, pg/mL	68	1.08 (1.19)	0.19	.12
IL-8, pg/mL	68	6.79 (5.18)	0.15	.22
Malondialdehyde, μM	68	4.80 (0.88)	−0.11	.36
Neurofilament light, pg/mL	68	50.77 (65.10)	0.03	.79
NT-proBNP, pg/mL	68	357.79 (388.68)	−0.07	.54
Oxidized LDL, U/L	68	40.96 (34.07)	0.69	<.001
Superoxide dismutase, U/uL	68	0.21 (0.06)	0.61	<.001
sTNFR-I, pg/mL	68	908.33 (253.13)	0.41	<.001
sTNFR-II, pg/mL	68	7159.88 (2308.42)	0.43	<.001
TNF, pg/mL	68	1.34 (0.49)	0.20	.10

Abbreviations: CRP, C-reactive protein; DHEA-S, dehydroepiandrosterone sulfate; IL, interleukin; LDL, low-density lipoprotein; NT-proBNP, N-terminal pro–brain natriuretic peptide; sTNFR, soluble tumor necrosis factor receptor; TNF, tumor necrosis factor.

SI conversion factors: To convert C-reactive protein to milligram per liter, multiply by 10; DHEA-S to micromole per liter, multiply by 0.027; NT-proBNP to nanograms per liter, multiply by 1.

Both BrainAGE score and cranial radiation dose were inversely associated with DHEA-S in female survivors who were younger than 10 years at diagnosis (eTables 3 and 4 in Supplement 1). This group had lower sex hormone precursor correlated with higher BrainAGE score (Spearman ρ = −0.44; *P* = .01) and higher cranial radiation dose (Spearman ρ = −0.48; *P* = .01). Cranial radiation dose was directly associated with sTNFR-I and sTNFR-II (Spearman ρ = 0.53 [*P* = .002] and ρ = 0.50 [*P* = .004]) and IL-6 (Spearman ρ = 0.37; *P* = .04), while BrainAGE score was directly associated with 8-hydroxyguanosine (Spearman ρ = 0.39; *P* = .03), homocysteine (Spearman ρ = 0.39; *P* = .03), and high-sensitivity CRP (Spearman ρ = 0.42; *P* = .02) in female survivors who were younger than 10 years at diagnosis and treated with cranial radiation. In females 10 years or older at diagnosis, IL-12p70 (Spearman ρ = 0.32; *P* = .048) was found to be directly associated with BrainAGE score, and oxidized LDL (Spearman ρ = 0.41; *P* = .01) was directly associated with cranial radiation dose. Among 14 adults (8 females) who were younger than 10 years at diagnosis, 6 (5 males and 1 female) had plasma biomarkers and received 40 Gy or higher cranial radiation dose (eTable 5 in Supplement 1). Glutathione peroxidase (Spearman ρ = 0.83; *P* = .04), IL-10 (Spearman ρ = 0.89; *P* = .02), and oxidized LDL (Spearman ρ = 1.00; *P* < .001) were significantly correlated with BrainAGE score in this group. Seven survivors (5 females) were younger than 3 years when treated with cranial radiation. However, no significant associations were found among age at cranial radiation treatment, BrainAGE score, biomarkers, or neurocognitive outcomes in adult survivors diagnosed before 3 years of age (eTable 6 in Supplement 1).

## Discussion

BrainAGE is a promising machine learning method for early detection of age-related structural brain changes associated with neurocognitive function and correlated with plasma biomarkers of oxidative stress, neuroinflammation, and cardiovascular health in childhood cancer survivors.^23^ These data suggest that chronic inflammation may affect brain structures and neurocognitive outcomes. Specifically, we found that protein biomarkers were correlated with brain aging in adult survivors of childhood cancer and that they differed from those seen in normal brain aging.^24^ We found direct correlations between cranial radiation dose and biomarkers of oxidative stress and chronic neuroinflammation a decade after completion of therapy in both male and female survivors. Protein biomarkers implicated in atherogenesis (homocysteine, 8-hydroxyguanosine, malondialdehyde, and oxidized LDL) were elevated in survivors with higher BrainAGE scores, suggesting that maintaining vascular health after cranial radiation may be crucial for preserving normal brain aging in childhood cancer survivors.^25,26^ Efforts could include reducing the risk of developing atherosclerotic disease with increased physical activity; good weight management; and potentially targeted interventions such as treatment of hyperhomocysteinemia with pyridoxine and/or folic acid, especially when using agents known to increase homocysteine levels (eg, metformin, niacin, and antiepileptics).^27^ Future endeavors could focus on the use of synthetic LOX-1 modulators that inhibit the upregulation of oxidized LDL, which are currently in development.^28^

Female survivors who were treated with high-dose cranial radiation before the age of 10 years had the most evidence of accelerated brain aging: we found a direct correlation between a marker of neuron damage and degradation (neurofilament light) and brain aging. Elevated neurofilament light is seen with normal brain aging and neurodegenerative diseases.^29,30^ However, multiple factors, such as heart disease, hypertension, metabolic stress, and sex hormone changes, can affect neurofilament light.^31^ The correlations between cranial radiation dose and lower estrogen precursor hormone (DHEA-S) levels, elevated biomarkers for heart disease (homocysteine and high-sensitivity CRP), and BrainAGE scores in females who were treated before 10 years of age suggest an association among estrogen production, cardiovascular disease, and accelerated brain aging in these survivors. A study of women diagnosed with multiple sclerosis found that treatment with 8-mg estriol (estrogen receptor agonist) reduced serum neurofilament light levels, indicating that even a weak estrogen hormone can be neuroprotective against neuronal damage.^32^ Overall, these findings suggest that ongoing neurodegenerative changes in female survivors may be precipitated by the absence of the protective properties of estrogen, coupled with increased risk for cardiovascular disease, leading to accelerated brain aging. However, because of the small number of female survivors in this subpopulation, caution is warranted in interpreting these findings.

For male survivors, correlations between cranial radiation, BrainAGE, and oxidative stress markers (oxidized LDL and malondialdehyde) and endothelial and neuroinflammatory markers (sTNFR-II) suggest a persistent endothelial inflammatory reaction decades after radiation exposure. Interestingly, sTNFR-II has been associated with increased risk of coronary heart disease in women but not men without cancer.^33^ This finding suggests that men treated with cranial radiation when they were children may have an additional cerebrovascular risk not seen in men without cancer. Male survivors may need additional intervention to reduce cardiovascular risk and may benefit from targeted interventions that aim to limit chronic inflammation by targeting sTNFR-II using novel peptide vaccines, which are currently under development.^34^

An inverse correlation of intrathecal methotrexate with BrainAGE score was seen in survivors who received cranial radiation without high-dose methotrexate. To put this finding in context, historically at St Jude Children’s Research Hospital, survivors who received both cranial radiation and intrathecal methotrexate were treated using Total Therapy protocols prior to 1980. These survivors received both a 24-Gy dose of cranial radiation and intrathecal methotrexate. As treatment evolved, cranial radiation doses were reduced to 18 Gy for standard risk and high-dose methotrexate and intensified intrathecal methotrexate were introduced. By the early 2000s, cranial radiation had been eliminated, with patients receiving pulses of high-dose methotrexate and intensified intrathecal methotrexate. To test for this well-known inverse correlation between intrathecal methotrexate and cranial radiation attributed to these historical protocols,^35^ we tested the 2 groups (with vs without cranial radiation) and did not observe protective properties in the intrathecal methotrexate group. This finding may imply that the addition of high-dose methotrexate negated the inverse correlation of cranial radiation with intrathecal methotrexate.

### Limitations

This study has several limitations that affect the interpretation of some findings. First, given the cross-sectional design, we were unable to establish a causal association between biomarker concentrations and BrainAGE scores. Moreover, we were only able to collect plasma biomarkers from 141 of the 235 survivors with neuroimaging assessments. We were also not able to analyze a subset of the biomarkers we selected for testing due to large variations in the assay coefficients. Thus, we did not assess the presence of reactive nitrogen species and cyclooxygenase-dependent inflammation or confirm the role of lipid peroxidation. Finally, the study population represents a select group of survivors from a single institution who were treated with historical treatment regimens. However, the population included a diverse group of diagnoses and treatments.

## Conclusions

The evidence presented in this cross-sectional study supports the hypothesis that survivors of childhood cancer who were treated with cranial radiation continue to produce biomarkers of oxidative stress and inflammation a decade after completion of therapy. Those biomarkers were elevated in survivors with structural brain changes consistent with accelerated brain aging. Moreover, we found that brain-aging changes were associated with poorer neurocognitive outcomes. The elevated biomarker associated with vascular injury and heart disease in both male and female survivors in this study suggests that the lasting impact of radiation may be mediated by vascular disease. Future research is needed to replicate our findings in a larger cohort using targeted interventions to reduce the risk of vascular disease in this at-risk population. Additionally, future studies could focus on the interaction between sex hormones in female survivors who receive cranial radiation and structural brain changes.

## Notes

Supplement 1. **eTable 1.** Description of Neurocognitive Domain, Main Neurocognitive Ability and Associated Measures **eTable 2.** Mean Z-Scores, Standard Deviation and Percent Impaired of Neurocognitive Domains in Community Controls and Survivors **eTable 3.** Spearman Correlation of Plasma Biomarkers and BrainAGE Between Female Survivors Who Were Less Than 10 Years and Those Who Were 10 Years Old or Greater at Time of Diagnosis **eTable 4.** Spearman Correlations of Plasma Biomarkers and Cranial Radiation Dose (Gy) Among Female Survivors Who Were <10 Years Old Compared With Those Who Were ≥ 10 Years of Age at Diagnosis **eTable 5.** Spearman Correlations of Plasma Biomarkers and BrainAGE Among Survivors Who Were <10 Years Old and Those Who Were ≥ 10 Years of Age at Diagnosis and Treated With ≥ 40Gy Cranial Radiation **eTable 6.** Statistics for Children <10 Years of Age at Diagnosis: BrainAGE, Protein Biomarkers, and Neurocognitive Outcomes Comparison Between Those Who Were Treated With Cranial Radiation Less Than 3 Years of Age and Those ≥3 Years of Age **eFigure 1.** Whisker Plot of Neurocognitive Outcomes Among Community Controls (Yellow) and Survivors (Green) **eFigure 2.** Scatter Plots of Neurocognitive Outcomes to BrainAGE Scores Among Cancer Survivors

Supplement 2.
Data Sharing Statement
