# Supplementary material for: Accelerated Brain Aging, Atherogenicity, and Neurocognition in Adult Survivors of Childhood Cancer
Source: JAMA Netw Open. 2025 Dec 30;8(12):e2551865. doi: 10.1001/jamanetworkopen.2025.51865 (PMC12754683; doi:10.1001/jamanetworkopen.2025.51865)
Supplement: Supplement 1. — eTable 1. Description of Neurocognitive Domain, Main Neurocognitive Ability and Associated Measures eTable 2. Mean Z-Scores, Standard Deviation and Percent Impaired of Neurocognitive Domains in Community Controls and Survivors eTable 3. Spearman Correlation of Plasma Biomarkers and BrainAGE Between Female Survivors Who Were Less Than 10 Years and Those Who Were 10 Years Old or Greater at Time of Diagnosis eTable 4. Spearman Correlations of Plasma Biomarkers and Cranial Radiation Dose (Gy) Among Female Survivors Who Were <10 Years Old Compared With Those Who Were ≥ 10 Years of Age at Diagnosis eTable 5. Spearman Correlations of Plasma Biomarkers and BrainAGE Among Survivors Who Were <10 Years Old and Those Who Were ≥ 10 Years of Age at Diagnosis and Treated With ≥ 40Gy Cranial Radiation eTable 6. Statistics for Children <10 Years of Age at Diagnosis: BrainAGE, Protein Biomarkers, and Neurocognitive Outcomes Comparison Between Those Who Were Treated With Cranial Radiation Less Than 3 Years of Age and Those ≥3 Years of Age eFigure 1. Whisker Plot of Neurocognitive Outcomes Among Community Controls (Yellow) and Survivors (Green) eFigure 2. Scatter Plots of Neurocognitive Outcomes to BrainAGE Scores Among Cancer Survivors [file jamanetwopen-e2551865-s001.pdf]

Supplemental Online Content

Phillips NS, Zhang S, Baedke J, et al. Accelerated brain aging, atherogenicity, and neurocognition in adult survivors of childhood cancer. *JAMA Netw Open*. 2026;9(1):e2551865. doi:10.1001/jamanetworkopen.2025.51865

eTable 1. Description of Neurocognitive Domain, Main Neurocognitive Ability and Associated Measures

eTable 2. Mean Z-Scores, Standard Deviation and Percent Impaired of Neurocognitive Domains in Community Controls and Survivors

eTable 3. Spearman Correlation of Plasma Biomarkers and BrainAGE Between Female Survivors Who Were Less Than 10 Years and Those Who Were 10 Years Old or Greater at Time of Diagnosis

eTable 4. Spearman Correlations of Plasma Biomarkers and Cranial Radiation Dose (Gy) Among Female Survivors Who Were < 10 Years Old Compared With Those Who Were ≥ 10 Years of Age at Diagnosis

eTable 5. Spearman Correlations of Plasma Biomarkers and BrainAGE Among Survivors Who Were < 10 Years Old and Those Who Were ≥ 10 Years of Age at Diagnosis and Treated With ≥ 40Gy Cranial Radiation

eTable 6. Statistics for Children < 10 Years of Age at Diagnosis: BrainAGE, Protein Biomarkers, and Neurocognitive Outcomes Comparison Between Those Who Were Treated With Cranial Radiation Less Than 3 Years of Age and Those ≥ 3 Years of Age

eFigure 1. Whisker Plot of Neurocognitive Outcomes Among Community Controls (Yellow) and Survivors (Green)

eFigure 2. Scatter Plots of Neurocognitive Outcomes to BrainAGE Scores Among Cancer Survivors

This supplemental material has been provided by the authors to give readers additional information about their work.

© 2026 Phillips NS et al. *JAMA Network Open*

eTable 1. Description of neurocognitive domain, main neurocognitive ability and associated measures.

Cognitive Domain	Ability	Measure
**Attention**	Sustained Attention	CPT Omissions
**Attention**	Attention Variability	CPT Variability
**Attention**	Attention Span	Digit Span Forward Total
		Digit Span Forward Longest Span Length
**Attention**	Focused Attention	Trails A
**Memory**	Short-Term Memory	CVLT Short-Delay Free Recall
**Memory**	Long-Term Memory	CVLT Long-Delay Free Recall
**Memory**	New Learning	CVLT Total for Trials 1-5
**Memory**	Visual Memory	Visual Selective Reminding
**Executive Function**	Flexibility	Trails B
**Executive Function**	Fluency	Verbal Fluency
**Executive Function**	Working Memory	Digit Span Backward Total
		Digit Span Backward Longest Span Length
**Executive Function**	Self-Monitoring	CPT Commissions
**Processing Speed**	Visuomotor Speed	Digit Symbol Coding
**Processing Speed**	Motor Speed	Grooved Pegboard Dominant Hand
**General Intelligence**	Verbal Reasoning	Vocabulary
**General Intelligence**	Non-Verbal Reasoning	Matrix Reasoning
**General Intelligence**	Full Scale IQ	Full Scale IQ
**Academics**	Word Reading	WJ Letter-Word Identification
**Academics**	Mathematics	WJ Calculation

© 2026 Phillips NS et al. *JAMA Network Open*

eTable 2. Mean z-scores, standard deviation and percent impaired of neurocognitive domains in community controls and survivors.

	**Community Controls**				**Survivors**			
	n	mean	SD	impaired (%)	n	mean	SD	impaired (%)
**Visuomotor speed**	43	0.53	0.90	6.98	245	-0.38	1.07	30.61
**Flexibility**	43	0.32	1.03	11.63	252	-0.72	1.72	35.32
**Motor speed**	43	0.05	0.95	13.95	251	-0.77	1.35	37.85
**Focused attention**	43	0.51	0.85	4.65	253	-0.15	1.43	20.16
**Word reading**	43	-0.05	0.60	4.65	237	-0.43	0.82	16.03
**Fluency**	43	0.40	1.18	18.60	251	-0.30	1.12	31.87
**Verbal reasoning**	43	0.14	0.93	9.30	251	-0.43	1.14	29.08
**Visual memory**	42	-0.10	1.03	28.57	205	-0.73	1.29	45.37
**Full scale IQ**	43	0.22	0.89	6.98	251	-0.27	1.08	22.31
**Attention span**	43	0.13	0.98	20.93	251	-0.37	1.05	33.07
**Attention variability**	43	0.29	0.99	6.98	241	-0.22	1.15	18.26
**Sustained attention**	43	0.25	0.68	9.30	242	-0.11	1.17	16.12
**New learning**	43	0.32	1.12	9.30	251	-0.14	1.24	20.72
**Working memory**	14	0.08	0.75	14.29	96	-0.45	1.09	33.33
**Impaired defined as a z-score 1 Standard Deviation below the mean (z-score = 0)**								

© 2026 Phillips NS et al. *JAMA Network Open*

eTable 3. Spearman correlation of plasma biomarkers and BrainAGE between female survivors who were less than 10 years and those who were 10 years old or greater at time of diagnosis.

Biomarker (units)	n	mean (SE)	rho	P value
Female survivors <10 years of age				
**8-Hydroxyguanosine (ng/mL)**	**31**	**7.61 (4.17)**	**0.39**	**0.03**
**DHEA sulfate (ug/mL)**	**31**	**1.43 (0.83)**	**-0.44**	**0.01**
Glutathione peroxidase (U/mL)	31	325.78 (94.64)	0.26	0.17
**Homocysteine (uM)**	**31**	**10.18 (2.70)**	**0.39**	**0.03**
**High sensitivity CRP (pg/mL)**	**31**	**8.08 (8.25)**	**0.42**	**0.02**
Interferon gamma (pg/mL)	31	11.97 (16.31)	-0.03	0.86
Interleukin-10 (pg/mL)	31	0.37 (0.28)	-0.14	0.46
Interleukin-12p70 (pg/mL)	31	0.15 (0.19)	-0.18	0.34
Interleukin-6 (pg/mL)	31	1.04 (0.96)	0.16	0.40
Interleukin-8 (pg/mL)	31	6.91 (3.59)	0.24	0.19
Malondialdehyde (uM)	31	4.55 (0.78)	0.14	0.36
**Neurofilament light (pg/mL)**	**31**	**63.05 (94.29)**	**0.37**	**0.04**
NT-proBNP (pg/mL)	31	294.21 (226.01)	0.23	0.20
Oxidized low-density lipoprotein (U/L)	31	65.12 (32.71)	-0.05	0.79
Superoxide dismutase (U/uL)	31	0.25 (0.04)	0.13	0.48
sTNFR-I (pg/mL)	31	958.44 (262.19)	0.00	1.00
sTNFR-II (pg/mL)	31	8119.86 (2576.54)	0.08	0.68
TNF alpha (pg/mL)	31	1.44 (0.53)	0.09	0.65
Female survivors ≥ 10 years of age				
8-Hydroxyguanosine (ng/mL)	39	12.87 (12.02)	0.13	0.44
DHEA sulfate (ug/mL)	39	1.83 (1.35)	0.20	0.23
Glutathione peroxidase (U/mL)	39	306.04 (70.79)	0.06	0.72
Homocysteine (uM)	39	11.71 (3.15)	0.04	0.81
High sensitivity CRP (pg/mL)	39	5.53 (6.46)	0.06	0.72
Interferon gamma (pg/mL)	39	13.58 (26.54)	0.06	0.70
Interleukin-10 (pg/mL)	39	0.32 (0.26)	0.00	0.98
**Interleukin-12p70 (pg/mL)**	**39**	**0.12 (0.14)**	**0.32**	**0.05**
Interleukin-6 (pg/mL)	39	1.24 (1.54)	0.02	0.89
Interleukin-8 (pg/mL)	39	6.52 (6.16)	-0.21	0.19
Malondialdehyde (uM)	39	5.03 (1.02)	0.11	0.50
Neurofilament light (pg/mL)	39	40.36 (15.13)	0.21	0.21
NT-proBNP (pg/mL)	39	395.67 (473.88)	0.00	0.99
Oxidized low-density lipoprotein (U/L)	39	20.00 (18.34)	-0.04	0.79
Superoxide dismutase (U/uL)	39	0.17 (0.05)	-0.06	0.69
sTNFR-I (pg/mL)	39	866.49 (236.30)	-0.22	0.17
sTNFR-II (pg/mL)	39	6503.60 (1809.54)	-0.18	0.27
TNF alpha (pg/mL)	39	1.30 (0.48)	0.05	0.76

© 2026 Phillips NS et al. *JAMA Network Open*

eTable 4. Spearman correlations of plasma biomarkers and cranial radiation dose (Gy) among female survivors who were < 10 years old compared those who were ≥ 10 years of age at diagnosis.

Biomarker (units)	n	mean (SE)	rho	P value
		Female survivors <10 years of age		
8-Hydroxyguanosine (ng/mL)	31	7.61 (4.17)	0.07	0.69
**DHEA sulfate (ug/mL)**	**31**	**1.43 (0.83)**	**-0.48**	**0.01**
Glutathione peroxidase (U/mL)	31	325.78 (94.64)	0.25	0.17
Homocysteine (uM)	31	10.18 (2.70)	0.35	0.05
High sensitivity CRP (pg/mL)	31	8.08 (8.25)	0.28	0.13
Interferon gamma (pg/mL)	31	11.97 (16.31)	-0.09	0.62
Interleukin-10 (pg/mL)	31	0.37 (0.28)	-0.23	0.22
Interleukin-12p70 (pg/mL)	31	0.15 (0.19)	-0.32	0.08
**Interleukin-6 (pg/mL)**	**31**	**1.04 (0.96)**	**0.37**	**0.04**
Interleukin-8 (pg/mL)	31	6.91 (3.59)	0.13	0.49
Malondialdehyde (uM)	31	4.55 (0.78)	0.25	0.18
Neurofilament light (pg/mL)	31	63.05 (94.29)	-0.02	0.90
NT-proBNP (pg/mL)	31	294.21 (226.01)	0.15	0.43
Oxidized low-density lipoprotein (U/L)	31	65.12 (32.71)	0.32	0.08
Superoxide dismutase (U/uL)	31	0.25 (0.04)	0.32	0.08
**sTNFR-I (pg/mL)**	**31**	**958.44 (262.19)**	**0.53**	**0.002**
**sTNFR-II (pg/mL)**	**31**	**8119.86 (2576.54)**	**0.50**	**0.004**
TNF alpha (pg/mL)	31	1.44 (0.53)	0.30	0.10
		Female survivors ≥ 10 years of age		
8-Hydroxyguanosine (ng/mL)	37	12.68 (12.27)	-0.22	0.19
DHEA sulfate (ug/mL)	37	1.86 (1.36)	0.20	0.25
Glutathione peroxidase (U/mL)	37	307.43 (70.64)	0.09	0.58
Homocysteine (uM)	37	11.79 (3.21)	-0.07	0.68
High sensitivity CRP (pg/mL)	37	5.63 (6.62)	-0.10	0.57
Interferon gamma (pg/mL)	37	13.91 (27.22)	0.01	0.95
Interleukin-10 (pg/mL)	37	0.32 (0.27)	0.04	0.80
Interleukin-12p70 (pg/mL)	37	0.12 (0.14)	0.15	0.37
Interleukin-6 (pg/mL)	37	1.12 (1.36)	0.02	0.91
Interleukin-8 (pg/mL)	37	6.68 (6.26)	0.08	0.64
Malondialdehyde (uM)	37	5.01 (0.93)	-0.22	0.18
Neurofilament light (pg/mL)	37	40.48 (15.47)	0.29	0.08
NT-proBNP (pg/mL)	37	411.06 (481.87)	-0.09	0.59
**Oxidized low-density lipoprotein (U/L)**	**37**	**20.72 (18.56)**	**0.41**	**0.01**
Superoxide dismutase (U/uL)	37	0.18 (0.05)	0.31	0.06
sTNFR-I (pg/mL)	37	866.36 (240.81)	0.13	0.43
sTNFR-II (pg/mL)	37	6355.57 (1710.83)	-0.04	0.81
TNF alpha (pg/mL)	37	1.25 (0.45)	-0.07	0.70

© 2026 Phillips NS et al. *JAMA Network Open*

eTable 5. Spearman correlations of plasma biomarkers and BrainAGE among survivors who were < 10 years old and those who were ≥ 10 years of age at diagnosis and treated with ≥ 40Gy cranial radiation. Bold indicates significant p <0.05.

Biomarker (units)	n	male	female	mean (SD)	rho	p.value
< 10 years of age at diagnosis						
8-Hydroxyguanosine (ng/ml)	6	5	1	8.07 (3.01)	-0.20	0.70
DHEA sulfate (ug/ml)	6	5	1	2.35 (1.59)	0.03	0.96
**Glutathione peroxidase (unit/ml)**	**6**	**5**	**1**	**294.32 (39.70)**	**-0.83**	**0.04**
Homocysteine (uM)	6	5	1	10.10 (2.14)	0.14	0.78
High sensitivity CRP (mg/L)	6	5	1	5.12 (7.93)	0.14	0.79
Interferon gamma (pg/ml)	6	5	1	7.34 (3.24)	-0.20	0.70
**Interleukin-10 (pg/ml)**	**6**	**5**	**1**	**0.80 (1.19)**	**-0.89**	**0.02**
Interleukin-12p70 (pg/ml)	6	5	1	0.20 (0.24)	-0.54	0.27
Interleukin-6 (pg/ml)	6	5	1	0.83 (0.64)	-0.03	0.96
Interleukin-8 (pg/ml)	6	5	1	93.54 (212.55)	-0.09	0.87
Malondialdehyde (uM/L)	6	5	1	4.45 (0.77)	-0.09	0.62
Neurofilament light (pg/ml)	6	5	1	117.85 (152.60)	0.66	0.16
NT-proBNP (pg/ml)	6	5	1	264.93 (199.35)	0.09	0.87
**Oxidized low-density lipoprotein (U/L)**	**6**	**5**	**1**	**66.74 (13.85)**	**1.00**	**<0.001**
Superoxide dismutase (unit/ul)	6	5	1	0.21 (0.07)	0.77	0.07
sTNFR-I (pg/ml)	6	5	1	1052.14 (202.35)	-0.49	0.33
sTNFR-II (pg/ml)	6	5	1	7914.13 (1036.62)	-0.14	0.79
TNF alpha (pg/ml)	6	5	1	1.60 (0.53)	-0.60	0.21
≥ 10 years of age at diagnosis						
8-Hydroxyguanosine (ng/ml)	6	3	3	7.80 (3.37)	-0.09	0.87
DHEA sulfate (ug/ml)	6	3	3	2.49 (1.32)	0.26	0.62
Glutathione peroxidase (unit/ml)	6	3	3	333.82 (128.79)	-0.14	0.79
Homocysteine (uM)	6	3	3	11.24 (3.40)	-0.03	0.96
High sensitivity CRP (mg/L)	6	3	3	4.84 (5.95)	-0.03	0.96
Interferon gamma (pg/ml)	6	3	3	19.85 (35.35)	0.71	0.11
Interleukin-10 (pg/ml)	6	3	3	0.43 (0.48)	0.66	0.16
Interleukin-12p70 (pg/ml)	6	3	3	0.11 (0.13)	0.14	0.79
Interleukin-6 (pg/ml)	6	3	3	0.88 (1.08)	0.60	0.21
Interleukin-8 (pg/ml)	6	3	3	6.51 (3.23)	0.37	0.47

© 2026 Phillips NS et al. *JAMA Network Open*

Malondialdehyde (uM/L)	6	3	3	3.83 (1.44)	0.09	0.47
Neurofilament light (pg/ml)	6	3	3	47.01 (25.61)	0.60	0.21
NT-proBNP (pg/ml)	6	3	3	172.05 (106.28)	0.71	0.11
Oxidized low-density lipoprotein (U/L)	6	3	3	43.53 (27.26)	-0.49	0.33
Superoxide dismutase (unit/ul)	6	3	3	0.20 (0.05)	-0.60	0.21
sTNFR-I (pg/ml)	6	3	3	1010.41 (280.07)	0.71	0.11
sTNFR-II (pg/ml)	6	3	3	7888.84 (3554.44)	0.31	0.54
**TNF alpha (pg/ml)**	**6**	**3**	**3**	**1.31 (0.57)**	**0.83**	**0.04**

© 2026 Phillips NS et al. *JAMA Network Open*

eTable 6. Statistics for children < 10 years of age at diagnosis. BrainAGE, protein biomarkers, and neurocognitive outcomes comparison between those who were treated with cranial radiation less than 3 years of age and those ≥ 3 years of age.

	Mean (SE)	P-values
BrainAGE	7.56 (6.29)	0.82
**Protein Biomarkers (units)**		
DHEA sulfate (ug/mL)	1.64 (1.24)	0.37
IL-6 (pg/mL)	0.92 (0.89)	0.87
sTNFR-I (pg/mL)	1022.06 (124.94)	0.17
sTNFR-II (pg/mL)	8327.30 (1709.85)	0.52
**Neurocognitive outcomes**		
Cognitive flexibility	-1.60 (1.45)	0.99
Motor processing speed	-0.87 (1.09)	0.45
Visual Memory	-0.94 (0.72)	0.96
Verbal reasoning	-1.01 (1.24)	0.39
Word reading	-0.73 (0.86)	0.56

© 2026 Phillips NS et al. *JAMA Network Open*

eFigure 1. Whisker plot of neurocognitive outcomes among community controls (yellow) and survivors (green). Clinically significant differences were defined as ± 0.5 SD from the mean = 0.

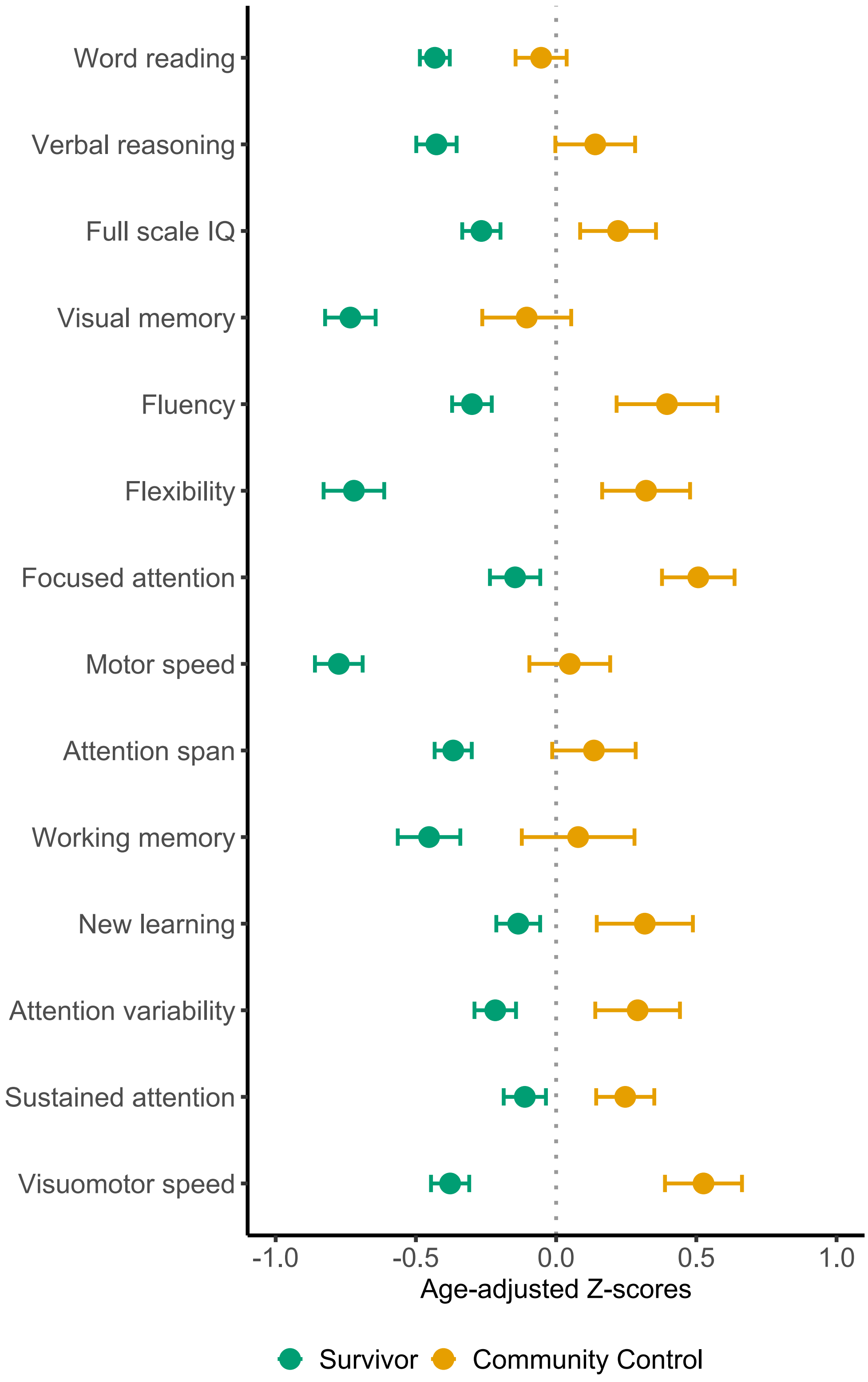

© 2026 Phillips NS et al. *JAMA Network Open*

eFigure 2. Scatter plot of neurocognitive outcomes to BrainAGE scores among cancer survivors. Greater difference between brain age and chronological age was associated with a worse performance in these domains. Example, a 10-year increase in BrainAGE score was associated with a 0.33 standard deviation drop in verbal reasoning z-score. Models were adjusted for age at diagnosis and sex. Red line indicates a linear regressor.

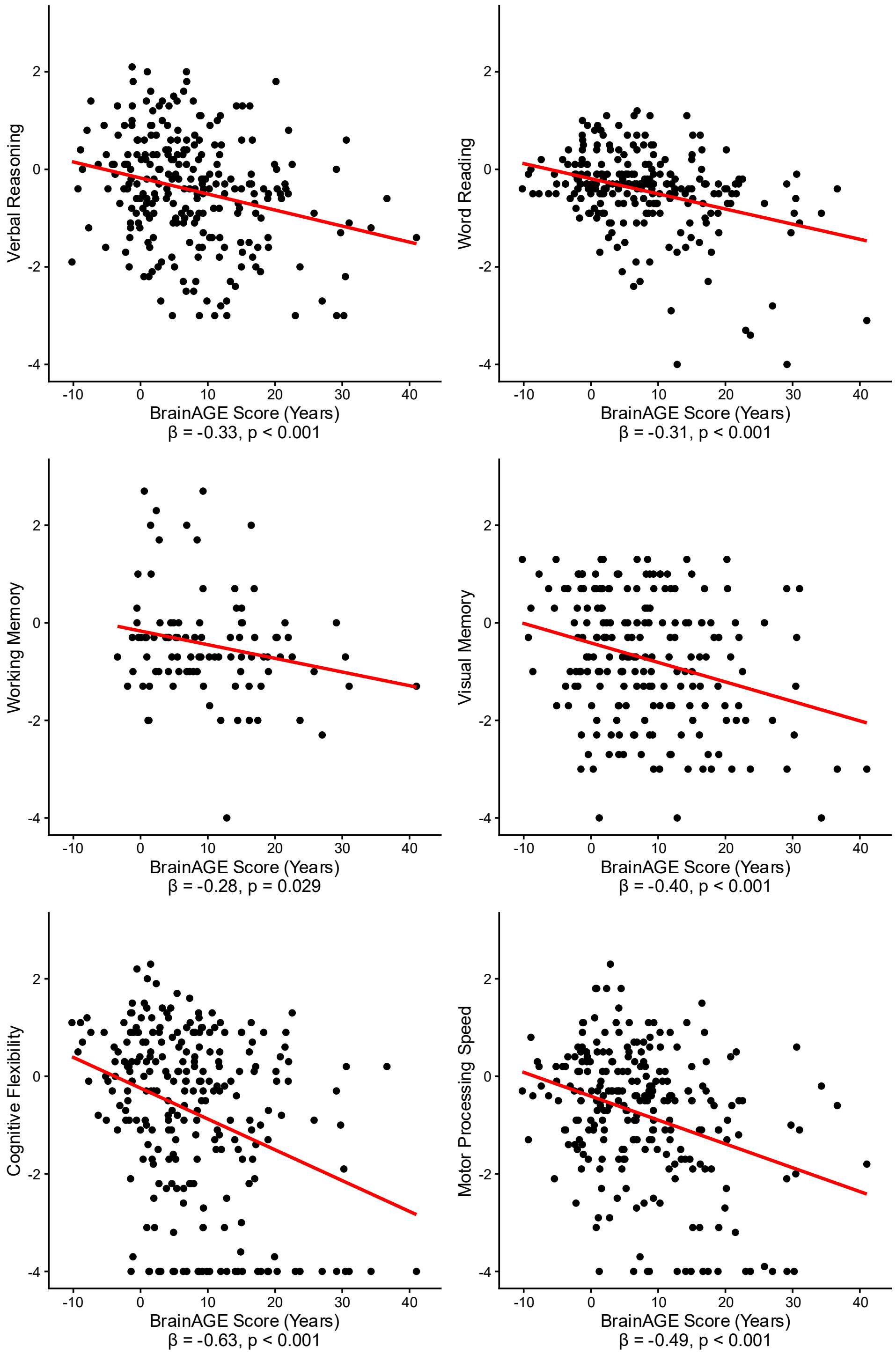

© 2026 Phillips NS et al. *JAMA Network Open*
