# Supplementary material for: Accelerated Brain Aging, Atherogenicity, and Neurocognition in Adult Survivors of Childhood Cancer
Source: JAMA Netw Open. 2025 Dec 30;8(12):e2551865. doi: 10.1001/jamanetworkopen.2025.51865 (PMC12754683; doi:10.1001/jamanetworkopen.2025.51865)
Supplement: Supplement 2. — Data Sharing Statement [file jamanetwopen-e2551865-s002.pdf]

Data Sharing Statement

Phillips. Accelerated Brain Aging, Atherogenicity, and Neurocognition in Adult Survivors of Childhood Cancer. *JAMA Netw Open*. Published January 02, 2026. doi:10.1001/jamanetworkopen.2025.51865

Data
Data available: Yes
Data types: Deidentified participant data, Data dictionary
How to access data: All data collected for this study is available to researchers outside of St. Jude to facilitate the development of treatments and cures for childhood cancer and its long-term effects. The data from this analysis will be posted on Zendo.com with the publication of this manuscript.
When available: With publication

Supporting Documents
Document types: None

Additional Information
Who can access the data: N/A
Types of analyses: All data collected for this study is available to researchers outside of St. Jude to facilitate the development of treatments and cures for childhood cancer and its long-term effects. The data from this analysis will be posted on Zendo.com with the publication of this manuscript.
Mechanisms of data availability: All data collected for this study is available to researchers outside of St. Jude to facilitate the development of treatments and cures for childhood cancer and its long-term effects. The data from this analysis will be posted on Zendo.com with the publication of this manuscript.
